# Air versus Water Chilling of Chicken: a Pilot Study of Quality, Shelf-Life, Microbial Ecology, and Economics

**DOI:** 10.1128/mSystems.00912-20

**Published:** 2021-03-02

**Authors:** Aeriel D. Belk, Toni Duarte, Casey Quinn, David A. Coil, Keith E. Belk, Jonathan A. Eisen, Jason C. Quinn, Jennifer N. Martin, Xiang Yang, Jessica L. Metcalf

**Affiliations:** a Department of Animal Sciences, Colorado State University, Fort Collins, Colorado, USA; b Department of Animal Science, University of California, Davis, California, USA; c Department of Mechanical Engineering, Colorado State University, Fort Collins, Colorado, USA; d Genome Center, University of California, Davis, California, USA; e Department of Evolution and Ecology, University of California, Davis, California, USA; f Department of Medical Microbiology and Immunology, University of California, Davis, Davis, California, USA; g CIFAR Azrieli Global Scholars program, CIFAR, Toronto, Canada; University of California San Diego

**Keywords:** chicken, meat, chilling methods, spoilage, shelf life, 16S rRNA gene, pseudomonas, techno-economics, energy, microbiome, 16S rRNA, chicken, technoeconomics

## Abstract

The United States’ large-scale poultry meat industry is energy and water intensive, and opportunities may exist to improve sustainability during the broiler chilling process. By USDA regulation, after harvest the internal temperature of the chicken must be reduced to 40°F or less within 16 h to inhibit bacterial growth that would otherwise compromise the safety of the product. This step is accomplished most commonly by water immersion chilling in the United States, while air chilling methods dominate other global markets. A comprehensive understanding of the differences between these chilling methods is lacking. Therefore, we assessed the meat quality, shelf-life, microbial ecology, and techno-economic impacts of chilling methods on chicken broilers in a university meat laboratory setting. We discovered that air chilling methods resulted in superior chicken odor and shelf-life, especially prior to 14 days of dark storage. Moreover, we demonstrated that air chilling resulted in a more diverse microbiome that we hypothesize may delay the dominance of the spoilage organism *Pseudomonas*. Finally, a techno-economic analysis highlighted potential economic advantages to air chilling compared to water chilling in facility locations where water costs are a more significant factor than energy costs.

**IMPORTANCE** As the poultry industry works to become more sustainable and to reduce the volume of food waste, it is critical to consider points in the processing system that can be altered to make the process more efficient. In this study, we demonstrate that the method used during chilling (air versus water chilling) influences the final product microbial community, quality, and physiochemistry. Notably, the use of air chilling appears to delay the bloom of *Pseudomonas* spp. that are the primary spoilers in packaged meat products. By using air chilling to reduce carcass temperatures instead of water chilling, producers may extend the time until spoilage of the products and, depending on the cost of water in the area, may have economic and sustainability advantages. As a next step, a similar experiment should be done in an industrial setting to confirm these results generated in a small-scale university lab facility.

## INTRODUCTION

Currently, the United States is the largest producer and second-largest exporter of poultry meat worldwide (1). The poultry industry in the United States has seen a fivefold production increase in the last 40 years and currently produces more than 50 million pounds of live birds annually (2). As a result of increased production, the poultry industry has also seen a tremendous rise in energy expenditures and water depletion (1). As demands for poultry meat continue to rise (3), novel approaches for reducing the environmental impact of poultry production, while not sacrificing poultry quality, need to be considered.

Temperature control during production and processing is a critical point in ensuring the safety and quality of poultry products. Thus, it is common for broiler production systems to reduce the internal temperature of chicken meat from 40°C to 4°C within 1 to 2 h following harvest. This step, though critical to maintaining the safety of the product, is time-consuming and requires significant investments in energy and water, depending on the chilling method utilized (4). Water immersion chilling (WC) and air chilling (AC) are the two most common chilling methods globally. Water immersion chilling is the most widely used chilling method in the United States, while AC is predominant in Europe, Brazil, and Canada (5, 6). During WC, eviscerated chicken carcasses are submerged in cold water that is often supplemented with antimicrobials intended to inhibit microbial growth. The application of these antimicrobials, combined with a continuous clean water system, results in notable reductions to the total bacterial population. However, cross-contamination, retained water on the carcass, consumer perception, water consumption, and wastewater management issues are a few challenges associated with WC (7–9). As mentioned, AC is widely utilized across Europe, Brazil, and Canada and involves the chilling of poultry carcasses by forced air in a cold room. Some studies have shown enhanced microbial quality and less exudative packaging in AC broilers compared to WC systems (7, 10). However, these microbial investigations were limited to culture-dependent techniques that focused on just a few microbes, and investigations with more robust sampling methods are warranted.

The rapid reduction in carcass temperature during poultry processing provides a unique thermodynamic challenge that requires significant energy inputs. Although AC results in a carcass with a slightly reduced yield (due to evaporative water loss), it has been estimated to require almost 50 times less gross energy than water chilling systems, when entire energy expenditures are considered (11, 12). In that regard, although WC methods are the most commonly utilized in the United States, the opportunity for significantly reducing water use and energy expenditures by converting to AC systems exists. However, before this transition can be made, it is imperative to assess how the conversion will affect the quality of the final product in addition to the economic viability of the production processing line.

To address meat quality, shelf-life, microbial, and techno-economic impacts of chilling methods, we conducted an experiment at the University of California, Davis (UC Davis) Meat Science Laboratory, in which chickens were chilled via either AC or WC. Novel to this experiment was the assessment of the impacts each chilling system had on the microbiome of poultry products and how that may relate to the quality of products from each system. This experiment yielded results that enhance the current knowledge regarding not only the quality of broiler meat produced using either chilling method but also the important techno-economics, which may guide industry investment or utilization in either system.

## RESULTS

### Experimental results.

In this experiment, 256 chicken carcasses were subjected to either air chilling (AC) or water immersion chilling (WC), then fabricated into either bone-in or boneless breasts before being placed under dark storage for either 7 or 14 days (see Fig. 5). At each step in the process, 10 carcasses or breasts per treatment were removed to collect physicochemical, microbial count, and microbiome data. These data were then further analyzed to determine the impact that the distinct processing methods had on the quality, shelf-life, and microbial ecology of chicken products.

The chilling method significantly impacted the carcass weight changes during the chilling process. On average, carcasses chilled using the WC system gained 5% of the prechilled weight, whereas carcasses chilled using the AC system lost 1.6% of the prechilled weight (*P* < 0.05; see Table S1 in the supplemental material). This difference in weight change is similar to what has been reported in other studies (12). There was no difference in pH between AC and WC chicken breasts (*P* > 0.05; data not presented).

10.1128/mSystems.00912-20.1 TABLE S1 Average weight loss of chicken carcasses prior to and following chilling by either air chilling (AC) or water chilling (WC). On average, the WC carcasses gained 5% of their prechilling weight, while AC carcasses lost 1.6% of their prechill weight. Download Table S1, TIF file, 1.0 MB. Copyright © 2021 Belk et al. 2021 Belk et al. https://creativecommons.org/licenses/by/4.0/ This content is distributed under the terms of the Creative Commons Attribution 4.0 International license.

### Quality and shelf-life implications of chilling strategy.

The shelf-life, or period until spoilage, was identified using the aerobic bacterial populations. Microbes were removed from the product surface using a rinsate, which was then serially diluted and plated on Petrifilm aerobic count plates (3M Microbiology, St. Paul, MN). Petrifilms were then incubated at 7°C and 35°C to obtain counts of psychrotrophic and mesophilic aerobic organisms, respectively. The WC chicken had fewer (*P* < 0.05) psychrotrophic bacteria (1.05 log CFU/g), organisms capable of growing at low temperatures, prior to fabrication (cutting of the chicken carcasses into breasts) than the AC chicken (2.12 log CFU/g). However, no difference in mesophilic bacteria, organisms that grow best at moderate temperatures, was observed between the two treatments for prefabrication samples (Table S2). On day 7, an approximately 1-log-unit difference in psychrotrophic bacteria was observed between AC (5.56 log CFU/g) and WC (6.59 log CFU/g) breasts, regardless of fabrication type (Table 1). WC and AC boneless breasts had lower total microbial counts throughout storage and display than the bone-in samples. Regardless, by day 14 of storage, chicken breasts from both chilling methods (WC and AC) and fabrication types (bone-in and boneless) had mesophilic aerobic bacteria populations greater than 7 log CFU/g, a threshold commonly associated with the end of shelf-life (13).

**TABLE 1:** Psychrotrophic bacterial counts for bone-in and boneless chicken breast cooled by either air chilling or water chilling at different time points^*a*^

Time	Psychrotrophic bacterial count (log CFU/g)^*b*^			
	Bone-in		Boneless	
	Air chilled	Water chilled	Air chilled	Water chilled
Prefabrication	2.12 BY (0.15)	1.05 AX (0.15)	2.12 BY (0.16)	1.05 AX (0.16)
Postfabrication	1.90 BY (0.16)	1.81 BY (0.15)	0.61 AX (0.16)	0.68 AX (0.16)
7-day storage (day 7)	5.86 CX (0.15)	6.96 DY (0.16)	5.26 CX (0.16)	6.22 DY (0.16)
End of display (day 10)	7.00 DY (0.15)	6.92 DY (0.15)	6.44 DY (0.16)	6.42 DY (0.16)
14-day storage (day 14)	8.71 EY (0.15)	9.00 EY (0.15)	8.51 EY (0.16)	8.67 EY (0.16)
End of display (day 17)	9.11 EY (0.15)	9.41 EY (0.16)	8.63 EY (0.16)	9.24 EY (0.16)

a Psychrotrophic bacterial counts for bone-in and boneless chicken breast cooled by either air chilling or water chilling pre- and postfabrication, pre- and postchilling, during storage, and during and after display.

b Least square means of psychrotrophic bacterial counts. Standard deviations are shown in parentheses. Values within a column with different letters (A to F) differ (*P* < 0.05). Values within a row with different letters (X and Y) differ (*P* < 0.05).

10.1128/mSystems.00912-20.2 TABLE S2 Least square means of mesophilic bacterial counts (log CFU/g) for bone-in and boneless chicken breast cooled by either air chilling or water chilling pre- and postfabrication, pre- and postchilling, during storage, and during and after display. Least square mean values within a column with different superscripts (a to e) differ (*P* < 0.05). D00, day 00. Download Table S2, TIF file, 1.5 MB. Copyright © 2021 Belk et al. 2021 Belk et al. https://creativecommons.org/licenses/by/4.0/ This content is distributed under the terms of the Creative Commons Attribution 4.0 International license.

**FIG 1:**
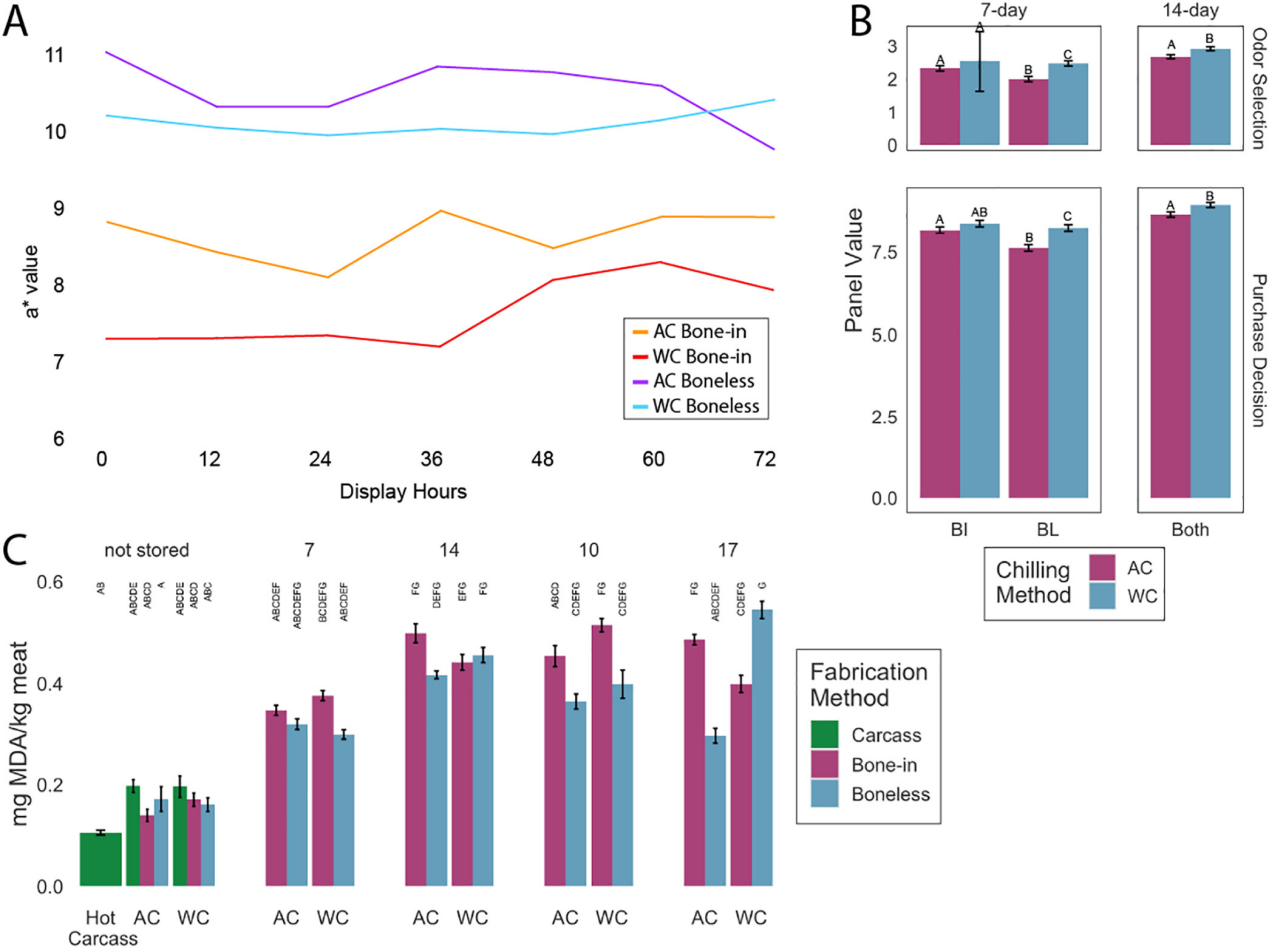
Changes in chicken quality over time. (A) Least square means of a* values for bone-in and boneless chicken breast chilled by either air chilling or water chilling following 7 days of dark storage, during 3 days of retail display. CIE a* represents the favorable redness of breasts. Chilling methods are represented by AC (air chilling) and WC (water chilling). (B) Least square means of consumer odor and purchase decision selection after dark storage and 3-day retail display. Fabrication methods are denoted as BI (bone-in) and BL (boneless). Breasts were placed in dark storage for either 7 or 14 days and then immediately placed in retail display. After 7 days of dark storage, an interactive effect was observed for both odor selection (*P* = 0.0132) and purchase decision (*P* = 0.0017). After 14 days, only the main effect of chilling methods was detected (*P* < 0.001). A hedonic three-point scale was used for the consumer odor selection (1 = desirable, 2 = acceptable, 3 = unacceptable) and purchase decision (7 = will buy, 8 = will buy with discount, 9 = will not buy). Bars in the same box with the same letter were not significantly different (*P* > 0.05). (C) The average lipid oxidation levels within a treatment group as measured by the thiobarbituric acid-reactive substance (TBARs) assay. Bars along the *x* axis refer to the chilling method and different colors represent the fabrication methods. As only chicken breasts were placed under dark storage, there were only carcass samples on the initial day of sample collection (not stored). Bars with the same letter were not statistically different (*P* > 0.05).

Instrument assessments of color were taken using a portable spectrophotometer (MiniScan EZ; Hunter Association Laboratory Inc., Reston, VA). These results demonstrated that the International Commission on Illumination (CIE) a* (redness) and b* (yellowness) values were greater (*P* < 0.05) for AC breasts than WC breasts throughout the display period, indicative of more desirable red and yellow tones within the muscle of AC breasts (Fig. 1A). Similarly, panelist evaluations indicated that the boneless chicken breasts were more desirable than bone-in breasts during the 3-day display period following 7 days of dark storage. Although there were notable differences in instrument color between the chilling methods, the difference was not observed in consumer preference. During the 3-day display following 14 days of dark storage, panelists considered the color and odor of all samples unacceptable regardless of the chilling method or fabrication type (Fig. 1B). Chilling method and fabrication type did not have an impact on texture selection (*P* > 0.05). Additionally, trained sensory panelists detected no differences in flavor or texture attributes between the chilling methods (*P* > 0.05) (Table S3).

10.1128/mSystems.00912-20.3 TABLE S3 Trained panelists were asked to evaluate chicken breasts for texture and flavor attributes on a 100-point scale. Within a column, values with the same letter were not significantly different (*P* > 0.05). Download Table S3, TIF file, 0.8 MB. Copyright © 2021 Belk et al. 2021 Belk et al. https://creativecommons.org/licenses/by/4.0/ This content is distributed under the terms of the Creative Commons Attribution 4.0 International license.

As expected, lipid oxidation increased as the time after chilling progressed (Fig. 1C). Lipid oxidation levels, as indicated by measurement of thiobarbituric acid-reactive substances, were similar across chilling method and fabrication type on the initial day of processing, 7 days of dark storage, 14 days of dark storage, and 7 days of dark storage with 3 days of retail display (*P* > 0.05), though there were differences when comparing sampling groups across these days. The greatest differences between treatments were seen on the samples collected after 14 days of dark storage with a 3-day retail display. Among samples from this time point, the boneless WC breasts had a higher degree of lipid oxidation compared to the boneless AC breasts (*P* < 0.05).

Nutritional content was very similar between chicken breasts regardless of chilling method (Table S4). Dry matter measurements ranged from 26.7% to 28.3%, with an overall mean of 26.13%. Within this narrow range, the only difference between treatment groups was between bone-in and boneless breasts collected on the initial day of experimentation. Chilling method, dark storage, and retail display did not impact dry matter content of the chicken breasts (*P* > 0.05). The same pattern was detected in moisture, as moisture and dry matter are inversely related. Measurements of ash and crude protein revealed a few differences between treatment groups, but these did not reveal significant patterns (Table S4). Crude fat values were, overall, low for all chicken products, ranging from 0.35% to 1.43% with a mean of 0.86%. Differences in these values were detected between fabrication types within a chilling method and sampling day (*P* < 0.05); carcass samples and bone-in breasts had higher crude fat content within almost all chilling methods and sampling days (*P* < 0.05; Table S4). However, no differences were observed between WC and AC (*P* > 0.05). Following this trend, the relative abundance of fatty acids was not grossly different between chilling methods on any sampling day. However, among fatty acids with less than 10% relative abundance, linoleate methyl ester (C_18_ and C_18:9c12c_) were more abundant in WC than AC breasts after 7 days of dark storage with 3 days of retail display (see Fig. S1 in the supplemental material).

10.1128/mSystems.00912-20.4 TABLE S4 Analysis of nutrient composition (proximate analysis) values. Data are organized first by chilling method, including hot carcass (HC), air chilled (AC) and water chilled (WC) samples. Within a day, data are then organized by sampling day, which includes not stored (day 0), 7 or 14 days of dark storage, and 3 days of retail display after removal from dark storage (day 10 and day 17). Within a day, data are organized by fabrication method, including unfabricated carcass, bone-in breasts, and boneless breasts. Values with the same letter within a column are not significantly different (*P* > 0.05). Download Table S4, TIF file, 0.8 MB. Copyright © 2021 Belk et al. 2021 Belk et al. https://creativecommons.org/licenses/by/4.0/ This content is distributed under the terms of the Creative Commons Attribution 4.0 International license.

10.1128/mSystems.00912-20.6 FIG S1 Relative abundances of fatty acids present in air chilled (AC) and water chilled (WC) chicken meat. (A) All fatty acids present in the samples. (B) The “rare” fatty acids present at <10% relative abundance. Download FIG S1, TIF file, 2.9 MB. Copyright © 2021 Belk et al. 2021 Belk et al. https://creativecommons.org/licenses/by/4.0/ This content is distributed under the terms of the Creative Commons Attribution 4.0 International license.

### Microbial ecology of chilled, fabricated, and packaged chicken.

The microbial ecology of the chicken products was investigated using 16S rRNA gene sequencing. Microbes were removed from the surfaces of the products using a sterile rinsate, from which DNA was extracted and sequenced following the Earth Microbiome Project protocols (https://www.earthmicrobiome.org/protocols-and-standards/16s/). Sequencing a total of 286 samples and controls resulted in a total of 3,837,564 demultiplexed reads. After denoising, quality filtering, read joining, and chimera removal via the DADA2 pipeline, 3,262,269 sequence reads (ranging from 1 to 54,662 sequences per sample with a mean of 13,418 sequences) were assigned to 774 amplicon sequence variants (ASVs). Subsequently, we filtered out ASVs that were taxonomically identified as representing mitochondria and chloroplasts, resulting in 3,261,944 sequence reads and 752 ASVs. The commercial positive-control sample (Zymo, Irvine, CA) resulted in the expected community of five microbes with no unexpected taxa, indicating no major contamination (Fig. S2). There were 24 negative/mock DNA extraction controls included in this sequencing run, which resulted in an average of 113 reads per negative control (range, 1 to 1,086) compared to 15,195 sequences per sample for DNA recovered from chicken rinsate (range, 3 to 51,660). This was considered an acceptable level for quality control. Additionally, samples were rarefied at 6,152 sequences for diversity analysis, which was well above the highest negative-control level. After rarefaction, all samples below this threshold were excluded from the diversity analysis, retaining 202 out of 259 rinsate samples. Before further analysis, negative- and positive-control samples were excluded from the experimental data set.

10.1128/mSystems.00912-20.7 FIG S2 Comparison of positive-control samples (ZymoBIOMICS D6300) and the standard, expected composition as described by the company. Download FIG S2, TIF file, 2.6 MB. Copyright © 2021 Belk et al. 2021 Belk et al. https://creativecommons.org/licenses/by/4.0/ This content is distributed under the terms of the Creative Commons Attribution 4.0 International license.

**FIG 2:**
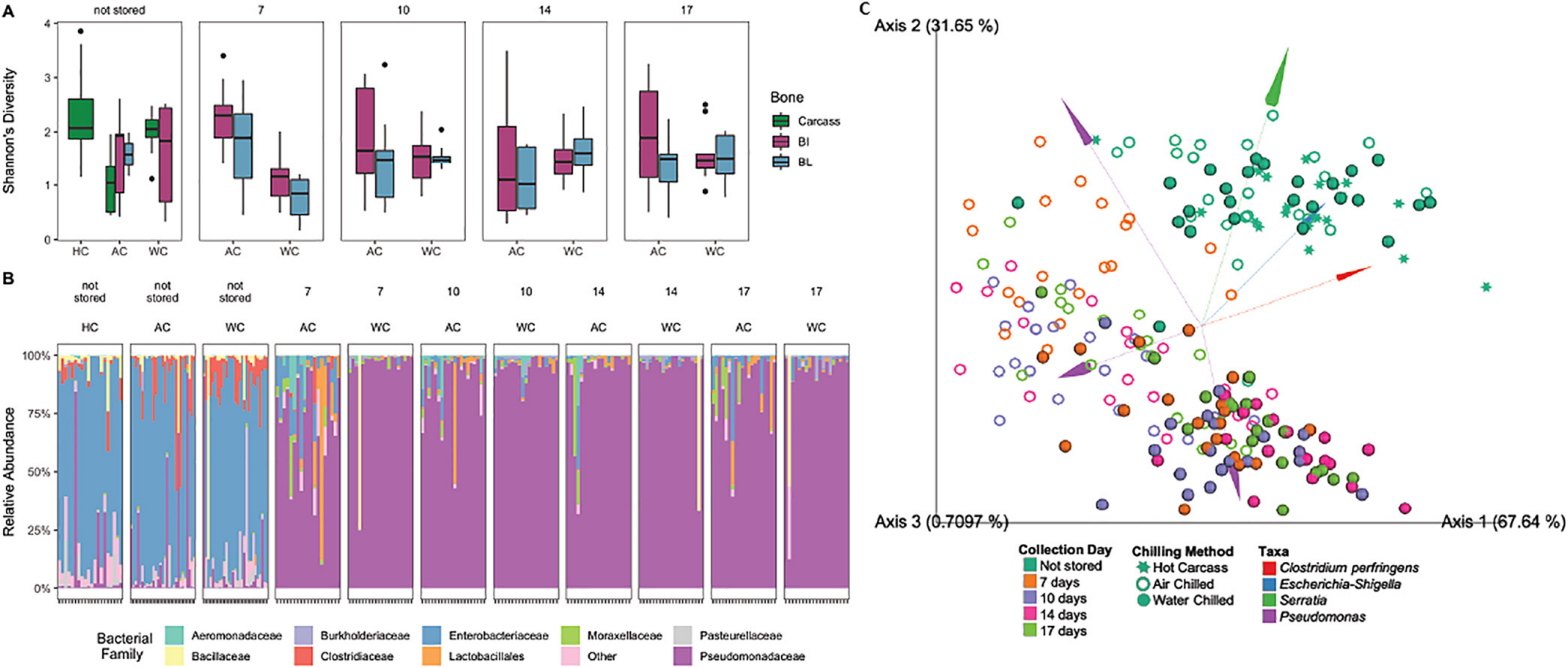
(A) Shannon’s diversity of the bacterial microbiome of chicken product samples, arranged by sampling day (7 and 14 days of storage plus 3 days of retail display) and chilling method (hot carcass [HC], AC, and WC) and colored by fabrication method (carcass, bone-in [BI], and boneless [BL]). The biomass of the WC-boneless samples was too low, resulting in few DNA sequences, and therefore, the samples were excluded after rarefying. (B) Taxonomy of the bacterial microbiome of chicken products based on analysis with the SILVA database, segmented by sampling day and chilling method. Within a facet, samples are organized as carcass, bone-in, and boneless. (C) A biplot constructed using robust Aitchison principal component analysis (PCA) that demonstrates separation in beta diversity between samples. Points are colored by the day samples were collected, including samples collected before dark storage (not stored), after 7 and 14 days of dark storage, and after 3 additional days of retail display (10 days and 17 days). The shape represents the chilling method, including hot carcass (prechill), air chilled, and water chilled. The lines show ASVs that are important to the direction of the biplot and are colored by the taxon associated with the specific ASV.

The alpha (within-sample) bacterial diversity of chicken products was reduced during chilling and processing (Fig. 2A). The hot carcass microbiome contained the highest mean alpha diversity (Shannon’s diversity = 2.29, Faith’s phylogenetic diversity = 9.96, observed ASVs = 50.6). During the chilling process, the mean Shannon’s diversity and observed ASVs were reduced, significantly (*P* < 0.05) in AC. After fabrication, or the cutting of the chicken carcasses into breasts, the alpha diversity increased, though not to the original levels. The high microbial diversity on chicken carcasses before product storage was associated with microbial communities dominated by organisms in the family *Enterobacteriaceae*. These communities also included bacteria at lower relative abundance from families *Clostridiaceae*, *Bacillaceae*, and *Pseudomonadaceae* (Fig. 2B). There were no significant differences in diversity between prestorage products (samples collected on the day of harvest that did not undergo a dark storage period) based on the chilling or fabrication method.

During storage and display, the alpha diversity of samples remained similar between treatment groups, while the beta diversity showed clusters separated by chilling method. The greatest difference was seen between AC and WC samples collected after removal from dark storage on day 7. At this time point, the diversity of the WC samples was lower (*P* < 0.05) than the diversity of AC bone-in samples and AC boneless samples. After the 7-day time point, the mean diversities were similar (Shannon’s diversity = 1.06 to 1.87, Faith’s phylogenetic diversity = 1.58 to 3.02, observed ASVs = 10.00 to 19.30). During these poststorage and postdisplay sampling points, *Pseudomonas* bacteria became the dominant group. For the chicken that was stored for 7 days, followed by 3 days of retail display, communities from WC chicken became dominated by *Pseudomonas* before AC chicken. When the beta diversity was calculated and visualized using robust Aitchison principal component analysis, samples separate initially by sampling day—all products that were stored, regardless of storage or display time, separated from samples that were not stored (Fig. 2C). Then, within the stored product, the samples clustered by chilling method. When the ASV vectors that explain these separations are overlaid and evaluated, it is clear that the main ASVs that separate the stored microbiome are *Pseudomonas* associated (Fig. 2C). Moreover, the separation between the chilling methods was primarily associated with distinct *Pseudomonas* ASVs. The patterns of changes in the microbial communities were predictive of spoilage and quality outcomes (Fig. S3). Using a Random Forest classifier, the microbiome could predict microbial spoilage, as defined by a psychrotrophic bacteria count of greater than 7 log CFU/ml, with an overall accuracy of 75%.

10.1128/mSystems.00912-20.8 FIG S3 Model prediction errors using the microbiome data as a predictor and spoilage indicators as response variables. The model predicting whether the product has reached a spoilage state was based on psychrotrophic bacterial counts. Model testing resulted in an accuracy score of 75%. Download FIG S3, TIF file, 0.6 MB. Copyright © 2021 Belk et al. 2021 Belk et al. https://creativecommons.org/licenses/by/4.0/ This content is distributed under the terms of the Creative Commons Attribution 4.0 International license.

### Phylogeny, diversity, and spoilage potential of *Pseudomonas*.

**FIG 3:**
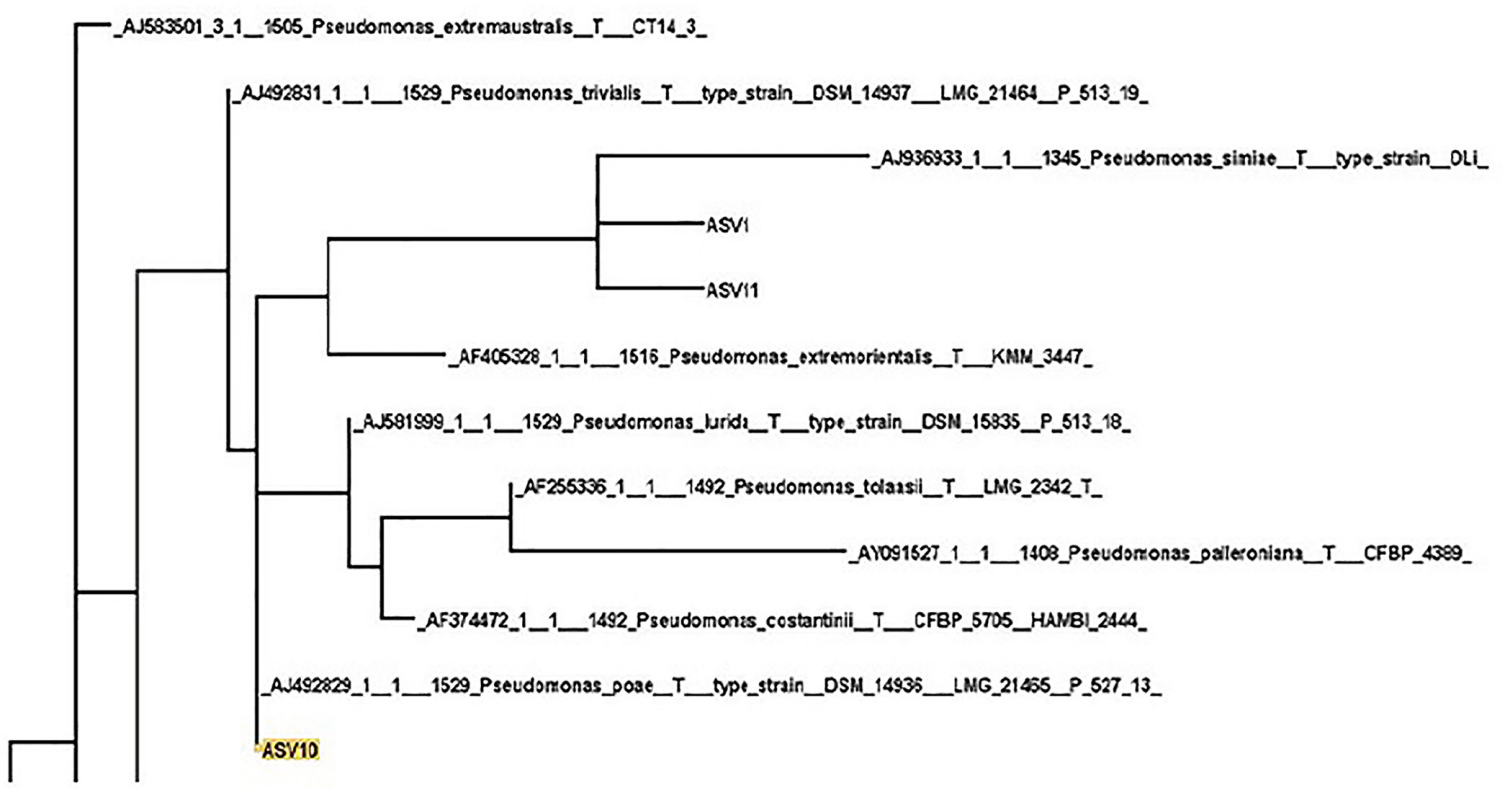
A portion of the detailed phylogenetic tree constructed from ASVs that were assigned to *Pseudomonas*. The larger tree is included in Fig. S2 in the supplemental material.

*Pseudomonas* ASVs (*n* = 33) come to dominate the microbial community during storage and display (Fig. 3), and due to the importance of these ASVs, we examined them in more detail. When placed into a phylogenetic tree containing 16S rRNA gene sequences from all *Pseudomonas* type strains, we reveal significant variation of branches associated with sequences from this genus. These ASVs are distributed throughout the phylogenetic tree and most likely represent multiple *Pseudomonas* species (Fig. S4). We also calculated the average percentage of reads in each sample that belonged to a *Pseudomonas* ASV (Table S5).

10.1128/mSystems.00912-20.5 TABLE S5 The most abundant *Pseudomonas-*associated ASVs associated with each chilling and storage group. The relative abundance of each ASV was averaged across samples within the group. Download Table S5, TIF file, 1.6 MB. Copyright © 2021 Belk et al. 2021 Belk et al. https://creativecommons.org/licenses/by/4.0/ This content is distributed under the terms of the Creative Commons Attribution 4.0 International license.

10.1128/mSystems.00912-20.9 FIG S4 Phylogenetic tree showing the evolutionary relationship between ASVs found in this study that were assigned to the *Pseudomonas* genus. Download FIG S4, TIF file, 1.9 MB. Copyright © 2021 Belk et al. 2021 Belk et al. https://creativecommons.org/licenses/by/4.0/ This content is distributed under the terms of the Creative Commons Attribution 4.0 International license.

In order to focus on particular ASVs, we performed an analysis of the composition of microbiomes (ANCOM) analysis at each sampling point (days 0, 7, 14, and 17) comparing the two chilling methods (pooling fabrication method). Prior to storage, none of the ASVs that were differentially abundant were ASVs assigned to *Pseudomonas*. At day 7, there was one *Pseudomonas* ASV which differed (ASV7; WC = 0.21%, AC = 14.6%), At day 10 (7 days with 3-day retail display), there was also one (ASV10; WC = 16.45%, AC = 1.8%); at day 14 there were three (ASV7, ASV10, and ASV20), and at day 17 (14 days with 3-day retail display) there were two (ASV7 and ASV20).

### Chilling system techno-economic analysis.

**FIG 4:**
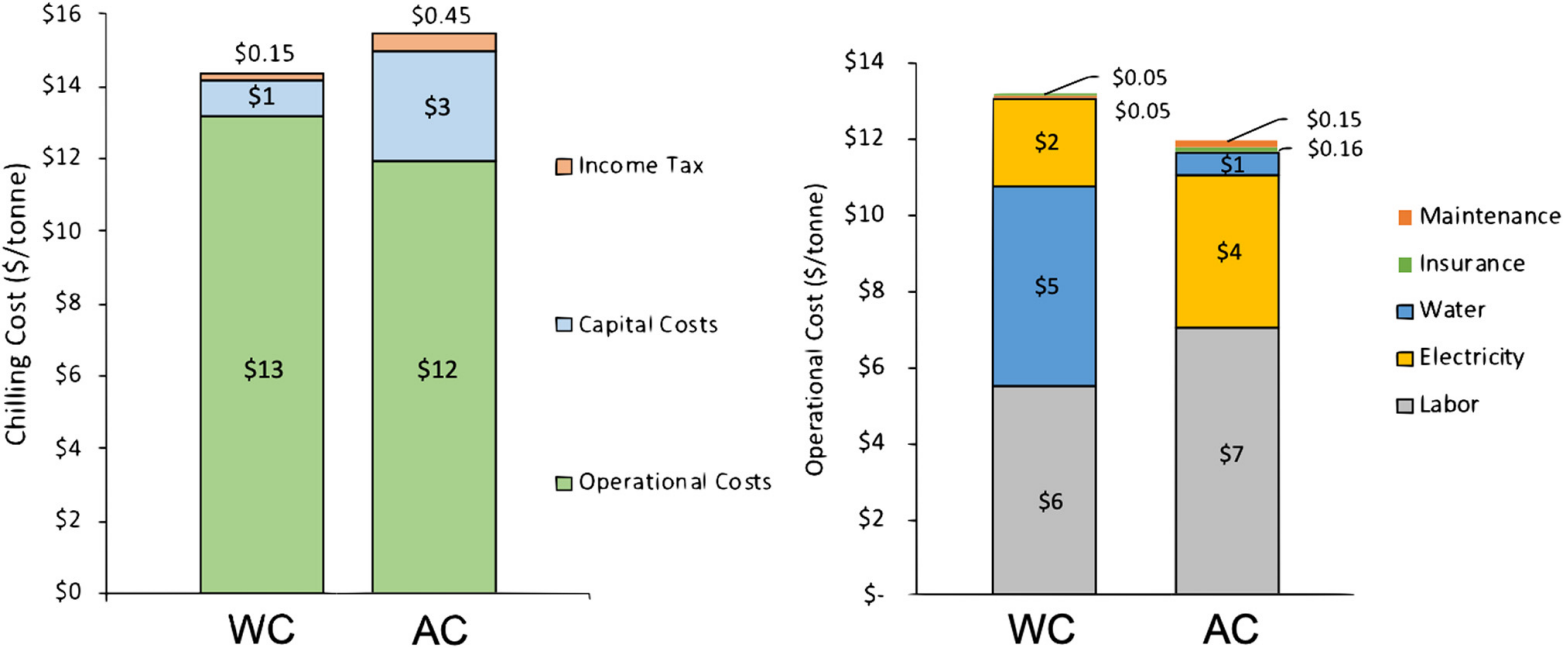
The levelized chilling cost (dollars/tonne or metric ton) for the baseline AC and WC models is shown in the left panel. The contributions of the capital costs, operational costs, and income tax to the total levelized chilling cost are displayed and demonstrate how the operational costs dominate the total chilling cost in both systems. The operational costs include the labor, electrical, water, maintenance, and insurance costs and are shown in the right panel for both the AC and WC systems.

Economic viability is a critical aspect of technology adoption. The levelized chilling cost (dollars/metric ton) for the baseline AC and WC models are shown in Fig. 4 with the total cost subdivided by capital cost, operational cost, and income tax. The costs for AC and WC are relatively similar, $15.45 per metric ton and $14.15 per metric ton, respectively. The operational costs dominate the total chilling cost for both the AC and WC systems. While the AC system has a higher capital cost than the WC system, the AC operational costs are less than the WC system. The primary difference between the two systems are the costs associated with electricity and water. The WC systems can use up to 10 times more water than the AC system, but the electrical costs of the AC system are only 2 times that of the WC system. Sensitivity analyses (Fig. S5) show there are scenarios where AC has the potential to be superior economically than WC, in particular for regions that might have lower electricity rates and high water purchase and treatment costs.

10.1128/mSystems.00912-20.10 FIG S5 The sensitivity analyses varied the following model values by ±50%: capital cost (dollars/tonne or metric ton), chilling floor space (m^2^), processing rate (metric ton/hour), chilling energy (kilowatt hour/metric ton), worker capacity (metric ton/hour), water use (liter/metric ton), water price plus water treatment cost (dollarsliter), and electricity cost (dollars/kilowatt hour). The resulting levelized costs for AC (upper panel) and WC (lower panel) systems demonstrate which aspects of each system have the most influence over the total levelized cost of chilling. For AC systems, capital costs and labor costs have the most impact on the total levelized chilling cost, while water and labor costs have the most impact on WC systems. Download FIG S5, TIF file, 1.8 MB. Copyright © 2021 Belk et al. 2021 Belk et al. https://creativecommons.org/licenses/by/4.0/ This content is distributed under the terms of the Creative Commons Attribution 4.0 International license.

## DISCUSSION

This study demonstrates that the method of chilling poultry carcasses not only influences the shelf-life and quality of chicken breasts but also has important implications for energy and water usage. Overall, in this study, chicken breasts from carcasses chilled using air chilling (AC) methods had superior odor and shelf-life (as assessed by psychrotrophic bacterial counts, consumer color and odor panels, and lipid oxidation patterns). Moreover, the microbial communities associated with AC products maintained diversity postchilling, and therefore may be more favorable by slowing the growth of spoilage organisms such as *Pseudomonas*. Finally, our techno-economic analysis highlighted potential economic advantages to AC compared to water chilling (WC) with advantages in areas of limited water and low-cost electricity.

Throughout dark storage and retail display, quality and shelf-life attributes were impacted by the chilling method. While all product was spoiled after 14 days of dark storage, notable quality and physical differences were observed among chicken treated with different chilling methods after 7 days of dark storage and 3 days of retail display. In this time period (7 days of dark storage to retail display), chicken chilled using AC demonstrated more desirable quality attributes, including more yellow tones and a lower abundance of spoilage-associated fatty acids, including linoleate methyl esters. These fatty acids have been associated with odor, color, and shelf-life challenges in previous studies (14–16). Differences in color and consumer appeal were similar to those reported by Jeong et al. (17), who demonstrated that while WC may reduce temperature more quickly, the use of AC resulted in superior color and juiciness. The color difference could be due to evaporative moisture loss drying the surface of the chicken, allowing for the breast muscle under the skin to become more visible (9). However, they differ from other studies that showed no difference in color between AC and WC breasts (18, 19). The spoilage patterns observed over time were similar to those previously reported by Katiyo et al. (20). Taken together, the color and fatty acid differences suggest that WC breasts may reach a spoilage state more rapidly than AC breasts.

We did not detect a significant impact on texture, flavor attributes, or nutritive composition by chicken chilling method. Previous studies have shown mixed impacts of chilling method on flavor and sensory attributes. For example, Hale et al. (21) suggested a flavor advantage in AC chicken compared to WC chicken, although their products were fried. Conversely, Ristic (22) suggested that WC can lead to flavor and texture advantages. The lack of consistent impacts on sensory and nutritive variables, combined with the similarities observed in our study, suggests that consumer eating satisfaction and nutrient composition would not be influenced by chilling method selection.

Our results suggest that the physical properties of water versus air may result in distinct initial chicken carcass microbial ecologies. Immediately after chilling we found lower psychrotrophic plate counts in WC than AC carcasses, which suggests that WC is physically washing more cells from the carcass than AC. Previously, Chen et al. demonstrated that water chilling alone reduced total viable counts while air chilling did not, which further supports this hypothesis (23). The difference in bacterial counts did not correspond to a difference in microbial diversity at this time point, similar to findings that demonstrated no difference in the presence of specific microorganisms between chilling methods (6, 24). These initial postchilling microbial communities were dominated by the families *Enterobacteriaceae*, *Clostridiaceae*, and *Bacillaceae*, in agreement with findings by Handley et al. (25). There were more obvious differences in the microbial assemblage patterns following 7 days of dark storage. At this point, there was no difference in psychrotrophic counts between AC and WC, but AC had a lower mesophilic count. These results are similar to those reported by Tuncer and Sireli (26), who concluded that AC was superior to WC in terms of pathogen growth, though they did not specifically investigate the spoilage bacteria described in the current study. Additionally, after the 7-day dark storage period, the AC microbial community was much more diverse than the WC community, which was dominated by *Pseudomonadaceae*. We hypothesize that by removing more bacterial cells during chilling, there is less microbial competition, which in turn allowed *Pseudomonadaceae* to grow more quickly in WC products. Competitive advantages associated with *Pseudomonas* were also found in Katiyo et al. (20). Furthermore, we observed more intersample diversity in the AC group than in the WC group. It is likely that, due to the nature of water chilling, there is more opportunity for the microbiomes to become homogeneous, while in air chilling the carcasses were kept separate, and therefore, microbes were not shared between carcasses. It has been well documented that water chilling can provide an opportunity for cross-contamination of pathogens, and these results suggest that this trend holds for all bacteria (27–30). However, there is evidence that this effect can be reduced or eliminated with the addition of antimicrobial compounds to the chill water, which was not done in this experiment (8). We hypothesize that the microbial ecology associated with AC delays the *Pseudomonas* bloom associated with spoilage and may extend the shelf-life.

The difference in microbial diversity of chicken breasts under different chilling methods was primarily due to different ASVs that were assigned to *Pseudomonas*, further demonstrating that the population of *Pseudomonas* is likely a major driver of the microbial community structure and spoilage outcomes. Our phylogeny of *Pseudomonas* ASVs strongly suggests that the high number of *Pseudomonas* ASVs represents real biological variation.

While *Pseudomonas* species, in general, are thought to cause food spoilage (31), they are not often identified to the species level. 16S rRNA sequencing was used in this study to estimate the microbes present in the products, which cannot always define microbial taxonomy at a species level. However, predictions of the *Pseudomonas* species present in chicken products were made by aligning ASV sequences to sequences extracted from the Ribosomal Database Project and maximum likelihood backbones generated using RAxML. These analyses suggest that a variety of distinct *Pseudomonas* sequences are present in the chicken carcass microbiome. Pseudomonas lundensis and Pseudomonas fragi are known to cause food spoilage in both dairy and meat (32–34) and were found in a clade with two of our ASVs (ASV24 and ASV27), but these ASVs were seen at similar abundance in both WC and AC samples (data not shown). Two other species of *Pseudomonas* known to be involved in spoilage, P. fluorescens and P. putida (32, 34), were not found in clades with any of the ASVs in this study. Furthermore, ASV7 (higher abundance in AC at days 7, 14, and 17), ASV21 (no difference), and ASV29 (no difference) were found in a clade with P. argentinensis, P. straminea, and P. punonensis which to our knowledge have not been previously shown to be involved in food spoilage. ASV10 (higher abundance in WC at days 10 and 14), ASV1 (no difference), and ASV11 (no difference) were found in a clade containing P. lurida, P. poae, P. trivialis, P. palleronia, P. tolaasii, P. costantinii, P. extremorientalis, and P. simiae. ASV20 (higher abundance in WC at days 14 and 17) and ASV14 (no difference) were found in a clade with P. veronii. While none of these latter *Pseudomonas* species has been directly shown to be involved in food spoilage, we hypothesize based on these data that at least some of them likely play a role in chicken spoilage.

Although our research suggests that AC may have shelf-life and quality advantages over WC, techno-economics are more nuanced. The WC systems can use up to 10 times more water than the AC system, but the electrical costs of the AC system are 2 times that of the WC system. Our baseline models do not support the claims by two other studies that AC systems require almost 50 times less gross energy than WC systems, when entire energy expenditures are considered (11, 12). Rather, our results agree with the conclusions of Northcutt and Smith, specifically, that WC and AC systems have similar total chilling costs (35). However, Northcutt and Smith also claimed that the large water requirements, which have been steadily increasing in the United States as a result of USDA regulations, can start to tip the balance for AC over WC systems especially if one considers the potential for changes in water purchase and treatment costs. Therefore, AC may have an economic advantage over WC depending on the local price and availability of water resources.

This experiment was designed to be a laboratory-based, pilot-sized representation of the larger process of chicken chilling, fabrication, storage, and retail display. We made efforts to represent industry conditions as accurately as possible in a small laboratory setting; however, there were a few conditions we were unable to replicate. We were unable to reproduce an antimicrobial application, either in the WC chill water or sprayed on the AC carcasses, which could modify some of the microbiological results. Therefore, our experiment represents a worse-case scenario. Moreover, all chicken carcasses used in this experiment were obtained from the same production lot in order to start all chilling processes at the same time. This may have led to a more homogeneous outcome than a more randomized selection. Future experiments should confirm the current findings using real industry conditions.

### Conclusions.

The overarching goal of this study was to combine multidisciplinary approaches to determine the impact of chilling method on the overall system efficiency and sustainability of chicken production. We were able to conclude that AC methods had an advantage in quality, spoilage, and consumer appeal prior to 14 days of dark storage, that AC appeared to result in a more favorable, diverse microbial community, and that AC requires less gross energy and, depending on the local price of water, may be the more economically favorable system.

## MATERIALS AND METHODS

### Experimental design.

The experiment was conducted using a 2 × 2 × 2 factorial design to evaluate three factors: chilling method (air chilling [AC] versus water immersion chilling [WC]), fabrication method (bone-in versus boneless), and dark storage period (7 days versus 14 days). Eviscerated, hot chicken carcasses (*n* = 256) were obtained from a commercial processing facility in California and transported to the USDA-inspected Meat Science Laboratory at the University of California, Davis (UCD) (Davis, CA) within 2 h of harvest. Carcasses were transported in sterile 150-quart coolers (MaxCold Cooler; Igloo Products Corp., Katy, TX) at a mean temperature of 30.25°C. Upon arrival at UCD, carcasses were divided into sampling groups following the scheme in Fig. 1. Sixteen carcasses were identified for a taste panel and placed four each in the treatment groups that were placed under 7-day dark storage (AC-bone-in, AC-boneless, WC-bone-in, WC-boneless). Of the other carcasses, 20 were sampled immediately for hot carcass samples, and the remainder were randomly and evenly assigned into either AC or WC (*n* = 110 birds/chilling method). Following chilling (described below), 10 carcasses from each AC and WC treatment group were sampled, and the remaining were evenly assigned to fabrication pathways (*n* = 50 birds/fabrication) yielding either bone-in or boneless chicken breasts (*n* = 20 breasts/fabrication pathway). Immediately following fabrication, 10 chicken breasts from each group were sampled, and the remaining breasts were placed on expanded polystyrene trays and overwrapped with polyvinyl chloride film (40-gauge; Berry AEP1504310). Overwrapped trays were placed in rigid cardboard boxes (*n* = 8 trays/box) and stored at 4°C (3 to 6°C) for either 7 or 14 days, a time frame that reflects industry standards. At each storage interval (7 or 14 days), individual packages of chicken breasts (bone-in and boneless) were removed from dark storage. Ten breasts from each group were sampled immediately after removal from storage, and the remaining packaged breasts were placed in a retail display case (Barker, Keosauqua, IA; average light intensity, 1,061 lux) maintained at 4°C (3 to 6°C) for 3 days.

### Chicken processing. (i) Procurement of chicken carcasses.

A commercial chicken processing facility in California was utilized to procure hot chicken carcasses for this research. Live birds were subjected to standard poultry harvest protocols as implemented by the commercial processing facility. Carcasses used for this experiment were obtained following defeathering, evisceration, and application of an initial postharvest antimicrobial carcass spray. Prior to chilling, the carcasses (*n* = 256) were removed from the processing line, placed in sterile plastic bags (*n* = 30 to 32 carcasses/bag), and bags were placed in sealed sterile coolers for transportation to the Meat Science Laboratory at University of California-Davis (UC-Davis) (Davis, CA). Additionally, temperature recorders (LogTag Tred30-16r; LogTag, Auckland, NZ) were placed in the coolers to monitor temperature during transportation.

### (ii) Processing and chilling of chicken carcasses.

**FIG 5:**
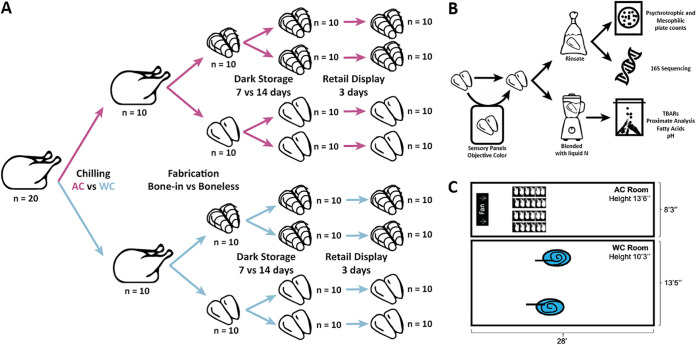
Representation of the experimental design. (A) The processing scheme with time points for sampling during the experiment. (B) The sampling process after the chicken product had been collected. The product was either sampled immediately or used for sensory panels. Microbial samples were taken via rinsate, which was then used for microbiological and microbiome analysis. The product was then flash-frozen in liquid nitrogen and powdered, then used for physicochemical analyses, including pH, thiobarbituric acid-reactive substance (TBARs) assay, proximate analysis, and fatty acid profiling. (C) Representation of the two rooms used for carcass chilling.

It should be noted that this process, while designed to mimic industrial systems, was performed on a much smaller scale. Upon arrival at the UC-Davis Meat Science Laboratory, 20 chicken carcasses were randomly selected for initial evaluations (described below), while the remaining carcasses were randomly and evenly assigned to one of two chilling methods (AC or WC; Fig. 5A). Weights (grams) of individual carcasses were obtained prior to chilling for comparison with weights obtained following chilling (described below). Sixteen carcasses were reserved for taste evaluation (described below) after 7 days of storage and 3 days of retail display. This subset of carcasses was subjected to both chilling methods and fabrication methods (described below), leaving 240 carcasses for laboratory analyses. Carcasses designated for WC were submerged in one of two simulated water chill tanks (Fig. 5C). Simulated water chill tanks were constructed from commercial water tanks (Structural Form Stock Tanks, 150 gallons; Rubbermaid), and a slurry of water and ice was formulated using potable water and commercial ice. Water temperature was monitored throughout chilling, and birds were agitated while submerged using a paddle. Carcass temperature was monitored regularly using a thermometer (Multitrip Data Logger; Temprecord, New Zealand) probe inserted into the thickest portion of the chicken breast. When the average internal carcass temperature reached 4°C, the chicken carcasses were removed from the water chilling system and placed on sterile wire racks for 10 min. Postchilling weight and temperature were obtained after the 10-min holding period. Additionally, five carcasses were randomly selected for analyses (described below).

To simulate air chilling, an isolated cold room in the UC-Davis Meat Science Laboratory was outfitted with a high-velocity fan (model BF60BDORGPRO; Maxx Air; 60-in. fan with 19,000 cubit feet per min [CFM], providing an air flow of 1.23 m/s) as shown in Fig. 5C. Chicken carcasses were placed on sterile wire racks located approximately 6 m from the commercial fan. The wire racks were rotated throughout the chilling process to ensure equitable exposure to the chilling conditions. Carcass temperature was monitored throughout by inserting a thermometer probe into the thickest portion of the breast, and once the average internal temperature reached 4°C, the carcasses were removed from the AC room. A postchilling weight and internal temperature were obtained from individual carcasses. Additionally, 10 carcasses from each method were randomly selected for analyses (described below).

### (iii) Fabrication of chicken carcasses, packaging of chicken breasts, and dark storage.

Immediately following chilling, carcasses within each chilling method (AC and WC) were randomly and evenly assigned to one of two fabrication methods (*n* = 50/fabrication method) for the generation of bone-in and boneless chicken breasts. Carcasses were fabricated, meaning cut from carcasses into individual parts, by trained personnel in the UC Davis Meat Science Laboratory using sterile instruments, and WC and AC carcasses were fabricated separately. Bone-in chicken breasts contained the ribs and part of the spine, while boneless breasts had these bones further removed. Immediately following fabrication, 10 chicken breasts from each group were sampled, and the remaining breasts were placed on expanded polystyrene trays and overwrapped with polyvinyl chloride film (40-gauge; Berry AEP1504310). Overwrapped trays were placed in rigid cardboard boxes (*n* = 4 trays/box) and stored at 4°C (3 to 6°C) for either 7 or 14 days.

### (iv) Retail display.

At each storage interval (7 or 14 days), individual packages of chicken breasts (bone-in and boneless) were removed from dark storage. Ten breasts from each group were sampled immediately after removal from storage, and the remaining packaged breasts were placed in a retail display case (Barker, Keosauqua, IA; average light intensity, 1,061 lux) maintained at 4°C (3°C to 6°C). Packages remained in the retail display case for 3 days. Instrumental meat color, measured via evaluating the lean color of the boneless samples and skin color of the bone-in samples, was taken using a portable spectrophotometer (MiniScan EZ; Hunter Association Laboratory Inc., Reston, VA) that was standardized before each use. A total of three readings of the International Commission on Illumination (CIE) L* (lightness), a* (redness), and b* (yellowness) values were taken using an illuminant A/10° observer for each breast. Measurements were taken through the packaging material at three separate locations on the chicken breast and were averaged prior to analyses. Packages were rotated in the display case every 12 h to ensure equitable temperature and light exposure.

### Microbial sample collection/processing.

At each sampling point, the microbial communities of the chicken products were collected using a rinsate method. At prefabrication time points, the entire chicken carcass was placed in a sterile collection bag (Whirl-Pak; Nasco, Fort Atkinson, WI) with 200 ml of phosphate-buffered saline (PBS) (National Diagnostics, Atlanta, GA) and agitated for 60 s to dislodge surface bacteria. After this, the carcass was removed from the rinsate and saved for physicochemical analysis. At the postfabrication time points, each chicken breast was divided in half. One half was placed in a sterile collection bag (Whirl-Pak; Nasco, Fort Atkinson, WI) with 50 ml of PBS and agitated for 60 s. The second half was reserved for physicochemical analysis. At all time points, the rinsate was collected from the sampling bag into 50-ml falcon tubes (Corning Science, Mexico) for analysis. A 10-ml aliquot of each sample was separated to be used for aerobic bacterial population analysis, and the remainder was frozen to –80°C and transported to Colorado State University (Fort Collins, CO) (Jessica L. Metcalf’s laboratory) for microbial ecology analysis.

The rinsate sample collected for microbiome analysis was further divided into 30-ml aliquots before DNA extraction. Cells within the rinsate were concentrated into a pellet by centrifugation at 4,600 × *g* for 15 min in a swing bucket rotor (Sorvall Legend X1R; Thermo Scientific, Waltham, MA). The supernatant was poured off, and a portion of the pellet equivalent to approximately 600 μl was used for analysis. DNA was extracted from the pellet following standard protocols utilizing the Qiagen PowerSoil DNA 96-well extraction kit (Qiagen, Hilden, Germany) following the manufacturer’s protocol for low biomass samples, which included the additional step of allowing the EB solution to be heated to 65°C before adding to the DNA plate wells for 5 min before eluting. DNA was eluted in two steps. Initially, 60 μl was eluted and considered our more concentrated DNA extraction. Next, an additional 80 μl of DNA was eluted. Each 96-well plate included eight mock extractions (no sample added) and one positive control (ZymoBIOMICS D6300). The 16S rRNA gene (V4 region) was amplified using primers 515F and 806R universal primers with the forward primer barcoded to allow for multiplexing during sequencing, following the Earth Microbiome Project protocols (https://www.earthmicrobiome.org/protocols-and-standards/16s/). The forward primer 515F included the unique sample barcode following Parada et al. (36), and both primers included degeneracies as described in Parada et al. and Apprill et al. (36, 37). Two PCRs using Invitrogen Platinum Hot Start PCR 2× Mastermix (Invitrogen, Carlsbad, CA) with 1 μl of DNA and a final concentration of 0.2 μM primer were run for each sample and combined to a total of 75 μl. The PCR product was quantified using a Pico Assay read by a Fluorskan plate reader (ThermoFisher Scientific, Waltham MA) and then pooled into a single pool in equimolar concentrations with the exception of samples that did not meet a minimum concentration, in which case 25 μl was added (this allowed the inclusion of mock extraction controls in the sequencing run). The resulting pool was cleaned using a Minelute PCR purification kit (Qiagen) and sequenced with a Miseq reagent v2 500 cycle kit at the Colorado State University (CSU) Next Generation Sequencing Core on the Illumina Miseq platform.

After sequencing, microbiome data were analyzed using QIIME2 (38) and R software version 3.5.1. Sequences were demultiplexed and quality filtered in QIIME2 using error-correcting Golay barcodes that prevent misassignment. Reads were trimmed to 250 bp, then amplicon sequence variants (ASVs) were inferred using DADA2 (39). Taxonomy was then assigned using the QIIME2 feature-classifier plugin (40) against the SILVA-132 99% database (41). Nonmicrobial sequences that assigned to mitochondria and chloroplasts were filtered from the data set. Samples were rarefied to 6,152 sequences, retaining 38.10% of features in 71.63% of samples, and diversity metrics were calculated using the QIIME2 core metrics pipeline. Statistical comparisons for alpha diversity were made using the Kruskal-Wallis test with an alpha diversity of 0.05, and statistical comparisons for beta diversity were made using permutational multivariate analysis of variance (PERMANOVA) with multiple testing correction and an alpha level of 0.05. The composition of the microbiomes was compared by testing the differential abundance of taxa using the ANCOM plugin in QIIME2 (42). The ability of microbial communities to predict quality and spoilage outcomes was assessed using the QIIME2 sample-classifier classify-samples plugin (43, 44). Models were trained and tested using k-fold cross-validation and the Random Forest classifier with hyperparameter tuning. Visualizations were generated using QIIME2 and R software with ggplot2 (38, 45).

### Phylogenetic trees.

All *Pseudomonas* ASV sequences were extracted from the feature table by filtering based on assigned taxonomy. An alignment of all type strain 16S rRNA gene sequences for this genus was downloaded from the Ribosomal Database Project (RDP) (46), along with an appropriate outgroup. A maximum likelihood backbone tree was generated using RAxML 8.2.12 (47) using the GTRGAMMA substitution matrix and 100 rapid bootstraps on the RDP alignment. An information file was then generated to be used in SATé-Enabled Phylogenetic Placement (SEPP) which was modified to fit the parsing parameters from pplacer v1.1.alpha13-0-g1ec7786 (removed one line according to documentation on the SEPP website [https://github.com/smirarab/sepp/issues/40]). SEPP 4.3.10 was then run with the following parameters (-P = 33 -A 10) to optimize the alignment breakdown using the ASV file, the RAxML tree, the RAxML info file, and the reference alignment as input.

### Quality measurements. (i) Aerobic bacterial populations.

As described by Martin et al. (64), aerobic bacterial populations are strong indicators of the end of shelf-life. Thus, quantifying the aerobic populations present—in addition to the characterization of the microbiome—will provide insight into the shelf-life impacts of the microbial population. At each sampling interval, the carcass of one sample from each chicken was rinsed using 200 ml PBS for the carcass and 50 ml for the breast. One milliliter of the rinsate was serially diluted in 0.1% buffered peptone water (BPW; Becton, Dickinson and Company, Sparks, MD) and plated in duplicate onto Petrifilm aerobic count plates (3M Microbiology, St. Paul, MN). Plates were then incubated at 7°C for 10 days and 35°C for 48 h.

### (ii) Physicochemical analysis.

Numerous biochemical and physicochemical changes that affect shelf-life occur in postharvest meat products (48). Thus, an assessment of these changes during processing was conducted to obtain information regarding product quality. At each sampling point, after the rinsate was collected, the sample was fabricated to a boneless breast if it was not already, though the skin was left on for carcass and bone-in samples. Then, the breast was flash-frozen in liquid nitrogen and homogenized using a blender (Magic Bullet; Capital Brands, Los Angeles, CA). To evaluate the carcass samples and bone-in breasts, the breast meat was removed from the bone at the time of sampling. The frozen homogenate was then transported to the Colorado State University Center for Meat Safety and Quality (Fort Collins, CO) for physicochemical and compositional analyses.

### (iii) Fatty acid composition.

Fatty acid composition was obtained using gas chromatography following methods described by Engle et al. and Kang and Wang (49, 50). First, fat was extracted using the Folch method (51). One gram of the frozen homogenate was combined with 20 ml of 2:1 chloroform-methanol mixture, homogenized, and then filtered using Whatman no. 1 filter paper (Fisher Scientific; Waltham, MA). Then, 4 ml of 0.9% NaCl solution was added per 20 ml of chloroform-methanol, and the solution was incubated at 4°C overnight. During this time the solvent separated into two phases; the lower phase contained the lipid extract, which was separated and dried in a dry matter oven at 100°C for 16 h. After this extraction, the lipid extract was methylated by adding 1 ml of 0.5 N KOH in methanol (MeOH) and heated in a water bath. Samples were then prepared for gas chromatography by mixing with 2 ml high-performance liquid chromatography (HPLC)-grade hexane and 2 ml saturated NaCl, which was then back-extracted and reconstituted to concentrate the fatty acids. The reconstituted lipid was measured by gas chromatography (Agilent 6890 plus; Agilent, Wilmington, DE) with standard fatty acid methyl ester mixtures and SUPELCO fatty acid methyl ester (FAME) standard (Millipore Sigma, Darmstadt, Germany) to calibrate. Fatty acids were identified by matching relative peak retention times to those of the standards, calculated as normalized area percentages of fatty acids.

### (iv) Lipid oxidation.

Lipid oxidation was measured using the thiobarbituric acid-reactive substance (TBARs) assay as described by Yin et al. (52). Briefly, 5 g of the frozen homogenate was mixed with trichloroacetic acid, homogenized using a standing homogenizer, and filtered using Whatman no.1 filter paper (Fisher Scientific, Waltham, MA). A 1-ml aliquot of the filtrate was mixed with 1 ml of 10 mM thiobarbituric acid and incubated at 25°C for 20 h, after which the absorbance at 532 nm was measured using a spectrophotometer (UV-2401; Shimadzu Inc., Columbia, MD).

### (v) Proximate analysis.

Nutrient composition analysis (proximate analysis) was conducted to determine the dry matter, moisture, ash, crude fat, and crude protein composition within each sample. Dry matter and moisture were measured using the AOAC oven drying method, 950.46 and 934.01 (53). Two grams of frozen homogenate was weighed, placed in a standard laboratory convection oven for 24 h at 60°C, and then reweighed. Percent moisture was calculated using the formula: % moisture content = [(wet weight − dry weight)/wet weight] × 100. Percent dry matter was calculated as 100 − moisture content. Ash content was determined using the ash oven method as described in the AOAC 923.03 and 920.153 (54). Approximately 1 g of the frozen homogenate was placed into a dry crucible and then inserted into a Thermolyne box furnace at 600°C for 18 h. Percent ash was calculated using the formula: % ash = (ash weight/wet weight) × 100. Crude fat was measured using the Folch method, as described above. Finally, crude protein was measured following AOAC method 992.15 (55), which used a TrueSpec CN nitrogen determinator (LECO, St. Joseph, MI). Percent protein was calculated using the formula: % protein = total % N × 6.25. Results were represented on a dry matter basis. Statistical analyses on all physiochemical tests were conducted using analysis of variance (ANOVA) and the emmeans package (56) with a 2 × 2 × 2 factorial design with an alpha level of 0.05.

### (vi) Sensory analysis.

Eight untrained participants were asked to evaluate the acceptability of three sensory attributes (color, odor, and texture) during retail display using a three-point sensory scale described by Lytou et al. (57). In addition, subjective color (desirable, acceptable, unacceptable) and willingness to purchase (would purchase, would not purchase, would purchase at a discounted price) was evaluated by these untrained panelists every 12 h during retail display. At the end of each 3-day retail display period (day 10 and day 17 ), the same participants were then asked to evaluate subjective odor, texture, and purchase selection using the same approach. Evaluation scores were analyzed as continuous data using mixed procedures of SAS (version 9.4; SAS Institute Inc., Cary, NC). Participants were treated as random variables, and the alpha level was defined as 0.05.

In addition to evaluation of chicken breast color, odor, and texture, trained taste panelists were asked to evaluate various palatability attributes (chicken flavor intensity, off-flavor intensity, springiness, cohesiveness of mass, and moistness) of bone-in and boneless chicken breasts representing both chilling methods following 7 days of dark storage. Panelists consisted of graduate students from the CSU Center for Meat Safety and Quality and were trained to recognize the aforementioned attributes using methods and references described by Solo (58). Samples for evaluation were randomized, and panels were conducted over 2 days to avoid sensory fatigue. Chicken breasts were cooked to an internal temperature of 76°C and cut into 2.54 × 2.54-cm cubes before being served to panelists under red lights. Panelists then ranked each breast portion for each of the above attributes on a 100-point scale. Data were analyzed using an ANOVA and the emmeans package in R (56) with an alpha level of 0.05.

### Chilling system techno-economic analysis.

An economic evaluation of each chilling system, AC and WC, was also performed. The work included the development of baseline models of each system that allowed for a direct comparison on the metric of economics. The baseline models used the same system boundaries that limited this techno-economic assessment to the chilling process and used harmonized model inputs when possible for consistency. Some facilities include maturation as an extension of the chilling process; however, these baseline models do not include anything outside the chilling process. The models used standard *n*th plant economic assumptions from literature and assumed a 10% internal rate of return (IRR), 20-year facility life, 8% loan interest rate on a 10-year loan with 40% equity, and the 2019 U.S. corporate tax rate of 21% (59–61). The above economic assumptions were combined with capital costs, operational costs, linear depreciation, and poultry processing rate to perform a 20-year discounted cash flow rate of return (DCFROR) for each poultry chilling system. These models use the IRR as the discount rate to determine the minimum processing cost (dollars/metric ton) associated with poultry chilling while providing a net present value (NPV) of zero. This minimum processing cost represents a levelized cost of chilling poultry carcasses that supports a 10% IRR over the 20-year life of the system.

All baseline values were taken from literature or acquired through communication with poultry chilling equipment manufacturers and poultry processing facilities. In particular, the system layout and energy consumptions reported in the literature were found to be antiquated, and thus, most of these data were acquired through communications with industry. The mutual baseline inputs were plant operation (250 days per year, 16 h per day), poultry processing rate (16.5 metric ton/hour), water treatment ($1.5/m^3^ [9, 35, 62, 63]), and electricity ($0.10/kilowatt hour) prices and fixed annual maintenance cost (5% of total capital cost). While AC and WC fixed and variable labor requirements might vary slightly, the labor requirements were assumed to be equivalent for the baseline cases for both systems. Based on input from chilling equipment suppliers, the AC and WC models reflect the major differences between the two systems (WC, AC); floor space requirements (100 m^2^, 500 m^2^), water use (3,200 liter/metric ton; 300 liter/metric ton), and chilling energy costs (20.9 kW h/metric ton, 31.4 kW h/metric ton). Due to the complexity and high variability in WC and AC system designs and operational characteristics, sensitivity analyses were used to evaluate how system parameters can impact the levelized cost associated with chilling poultry carcasses. The sensitivity analysis varied the following model values by ±50%: capital cost (dollars/metric ton), chilling floor space (square meters), processing rate (metric ton/hour), chilling energy (kilowatt hour/metric ton), worker capacity (metric ton/hour), water use (liter/metric ton), water price plus water treatment cost (dollars/liter), and electricity cost (dollars/kilowatt hour). The end result is a direct comparison of the two technologies in terms of costs with sensitivity used to highlight high impact variables.

### Ethics approval and consent to participate.

Human participation in taste and food quality evaluation was reviewed by the Institutional Review Board at Colorado State University with IRB ID 364-18H and declared to be exempt from the requirements of the human subject protection regulations.

### Data availability.

16S rRNA gene sequencing data are available in the EBI-ENA database, accession number PRJEB41700, and in QIITA, study 12193. Analysis details can be accessed at https://github.com/Metcalf-Lab/Air-versus-water-chilling-of-chicken.
