# Supplementary figures and images for: Air versus Water Chilling of Chicken: a Pilot Study of Quality, Shelf-Life, Microbial Ecology, and Economics
Source: mSystems. 2021 Mar 2;6(2):e00912-20. doi: 10.1128/mSystems.00912-20 (PMC8546986; doi:10.1128/mSystems.00912-20)

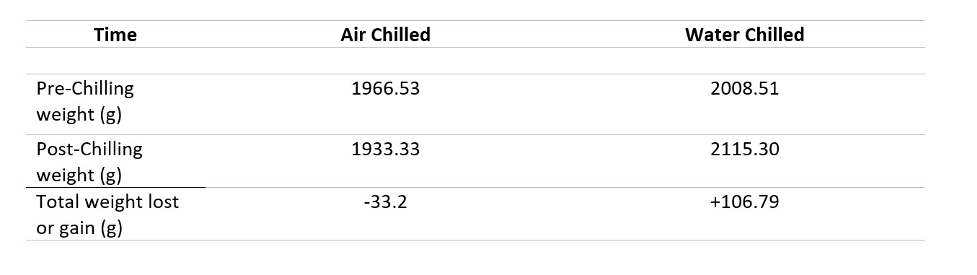

Supplement: TABLE S1 [file msystems.00912-20-st001.tif]

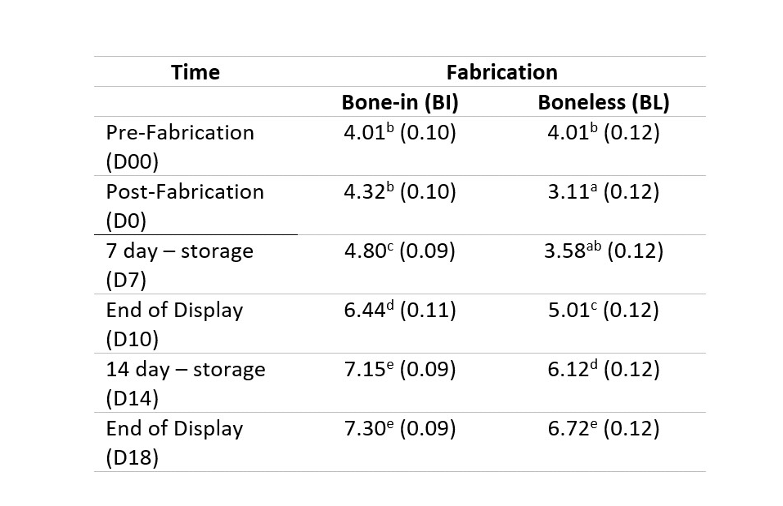

Supplement: TABLE S2 [file msystems.00912-20-st002.tif]

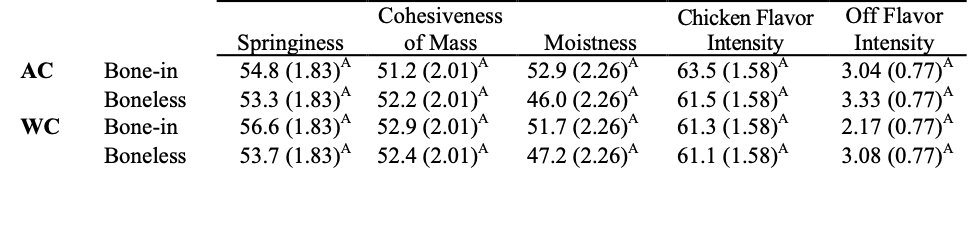

Supplement: TABLE S3 [file msystems.00912-20-st003.tif]

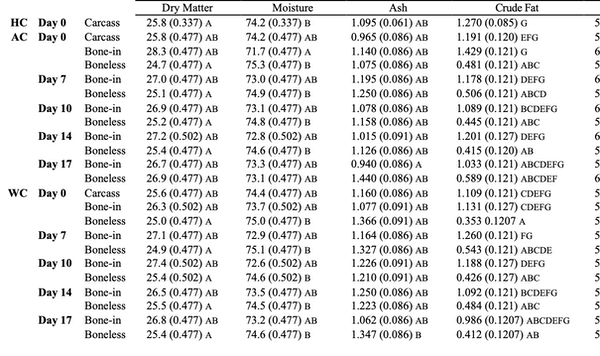

Supplement: TABLE S4 [file msystems.00912-20-st004.tif]

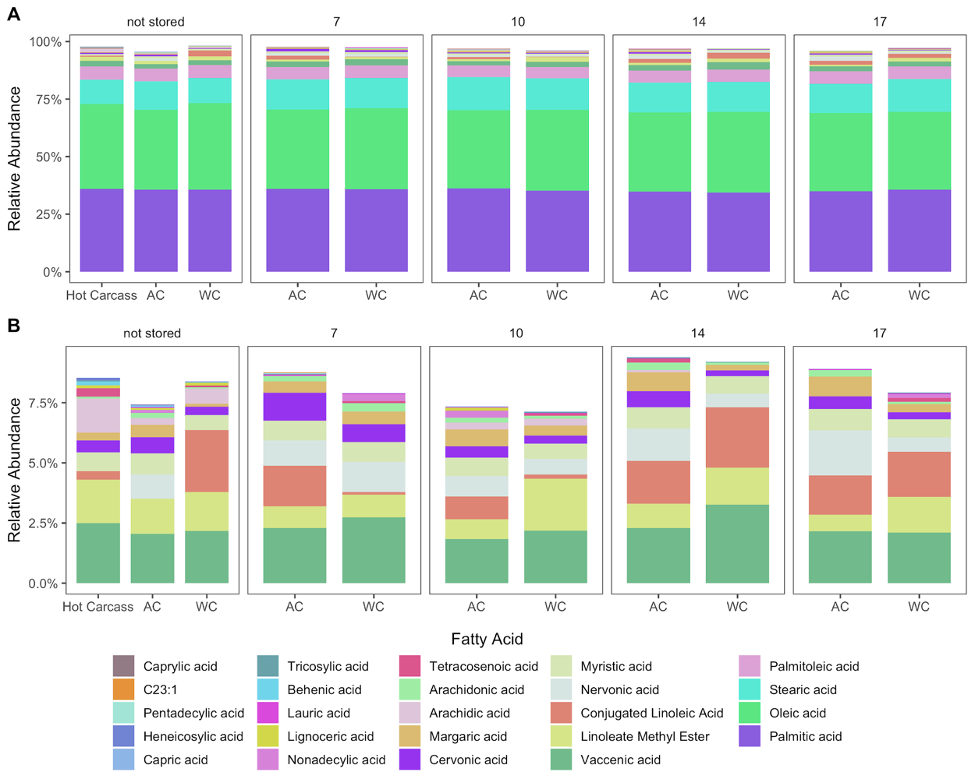

Supplement: FIG S1 [file msystems.00912-20-sf001.tif]

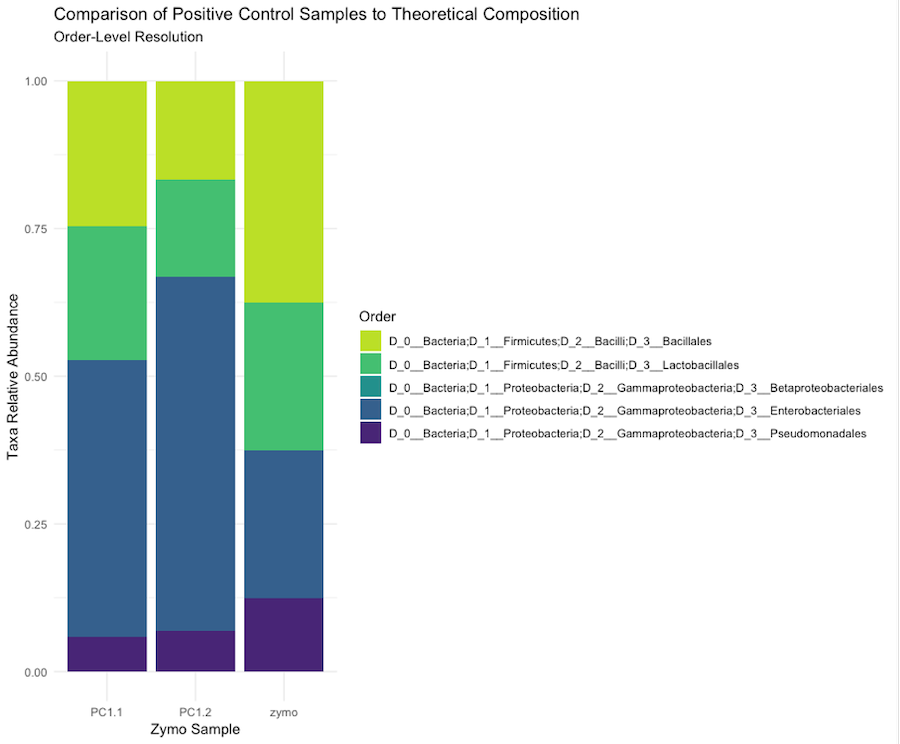

Supplement: FIG S2 [file msystems.00912-20-sf002.tif]

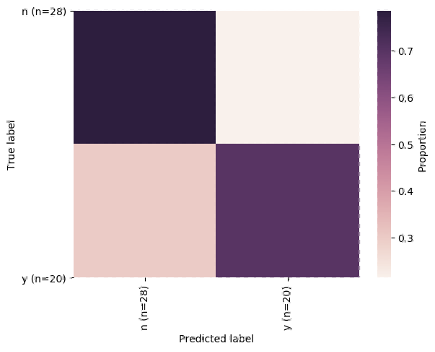

Supplement: FIG S3 [file msystems.00912-20-sf003.tif]

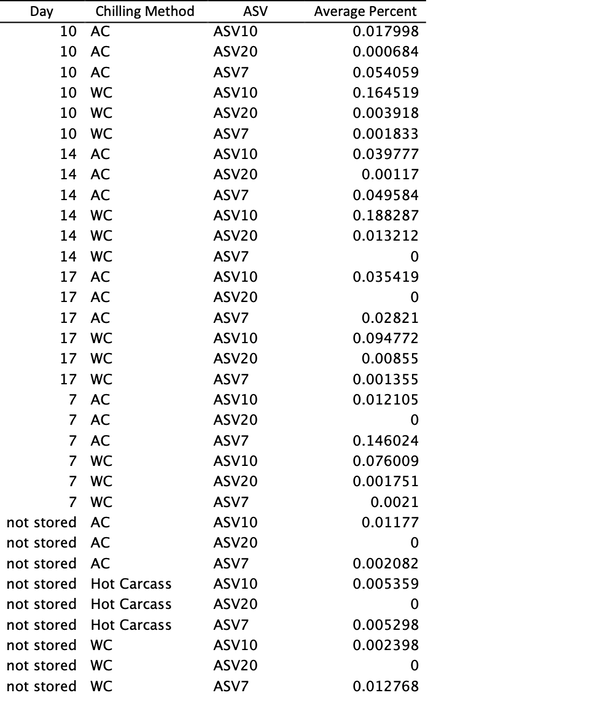

Supplement: TABLE S5 [file msystems.00912-20-st005.tif]

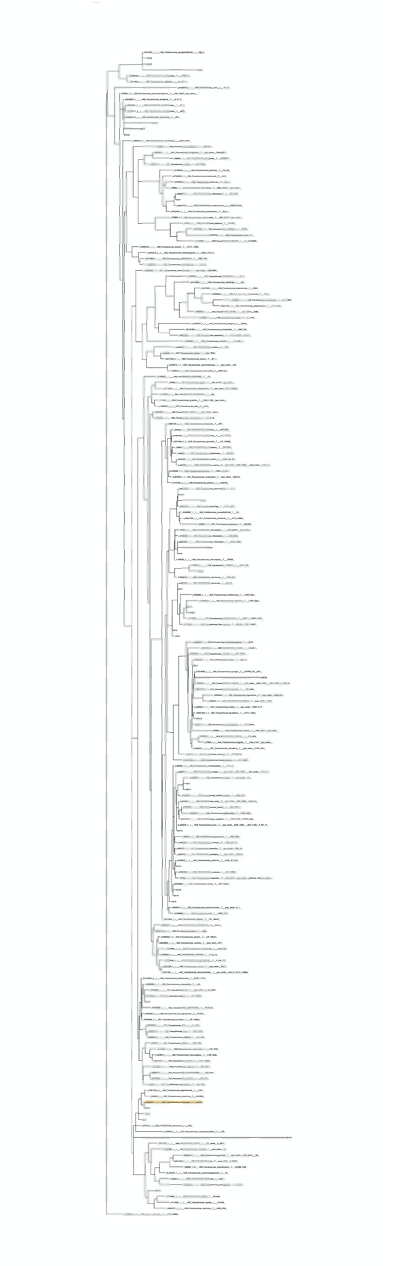

Supplement: FIG S4 [file msystems.00912-20-sf004.tif]

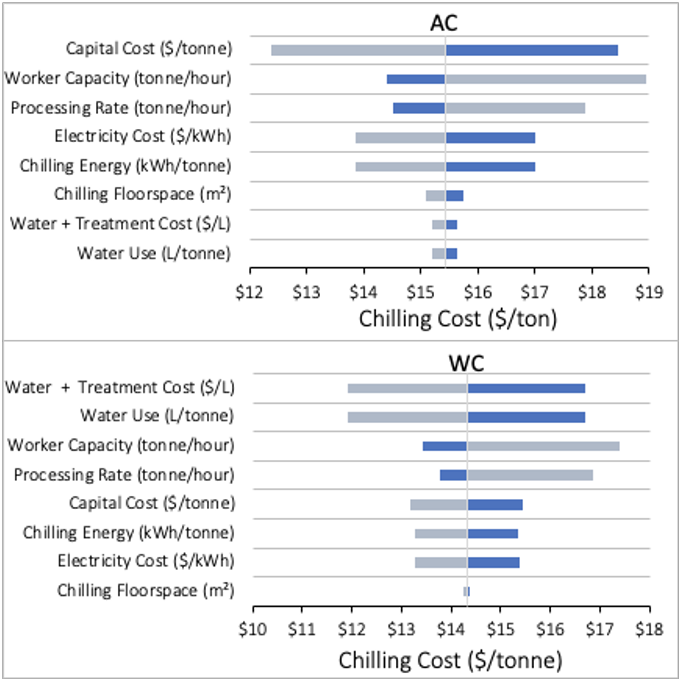

Supplement: FIG S5 [file msystems.00912-20-sf005.tif]
